# Microstructure Evolution and Damage Mechanism of DD9 Single Crystal Superalloy-Thermal Barrier Coating System Under High Temperature Oxidation: A Comparative Study with DD6

**DOI:** 10.3390/ma18184332

**Published:** 2025-09-16

**Authors:** Pan Li, Zhenyu Xin, Fan Sun, Xiaochao Jin, Chao Zhang

**Affiliations:** 1 School of Civil Aviation, Northwestern Polytechnical University, Xi’an 710072, China; lp0816@nwpu.edu.cn; 2 Key Laboratory on the Impact Protection and Safety Assessment of Civil Aviation Vehicle, Taicang 215400, China; 3 Xi’an Key Laboratory of Extreme Environment and Protection Technology, School of Aerospace Engineering, Xi’an Jiaotong University, Xi’an 710049, Chinajinxiaochao@xjtu.edu.cn (X.J.); 4 National Key Laboratory of Energetic Materials, Xi’an Modern Chemistry Research Institute, Xi’an 710065, China; sf2038945624@163.com; 5 School of Aeronautics, Northwestern Polytechnical University, Xi’an 710072, China

**Keywords:** thermal barrier coating, single-crystal superalloy, high-temperature oxidation, microstructural evolution, damage mechanism

## Abstract

This study investigates the microstructural evolution and damage mechanisms of the nickel-based single-crystal superalloy DD9-thermal barrier coating (TBC) system under 1050 °C high-temperature oxidation, while conducting a comparative analysis of oxidation behavior with the DD6-TBC system. Results show that both systems have similar oxidation mechanisms but face long-term oxidation drawbacks: as oxidation time increases, the thermally grown oxide (TGO) evolves into a mixed oxide layer and an Al_2_O_3_ layer, with initial rapid TGO growth consuming Al in the bond coat (BC) and subsequent Al depletion slowing growth, though long-term TGO accumulation raises cracking and spallation risks. DD9 and DD6 substrates significantly affect substrate-BC interfacial interdiffusion: the interdiffusion zone (IDZ) and secondary reaction zone (SRZ) grow continuously (SRZ growing faster), and linear topologically close-packed (TCP) phases precipitate in the SRZ, spreading throughout the substrate and impairing high-temperature mechanical properties. Specifically, DD9’s IDZ growth rate is faster than DD6’s in the first 800 h of oxidation but slows below DD6’s afterward, reflecting DD9’s superior long-term oxidation resistance due to better temperature resistance and high-temperature stability. This study clarifies key high-temperature service disadvantages of the two systems, providing experimental support for coated turbine blade life evaluation and a theoretical basis for optimizing third-generation single-crystal superalloy-TBC systems to enhance high-temperature service stability.

## 1. Introduction

The turbine inlet gas temperature is a critical factor determining the efficiency and performance of an aircraft engine [1,2]. To increase the turbine inlet gas temperature and, in turn, improve the thrust-to-weight ratio, advanced turbofan engine turbine blades commonly utilize nickel-based single-crystal superalloys as the base material, coupled with advanced cooling technologies [3,4]. Additionally, thermal barrier coating (TBC) is widely applied for heat insulation and oxidation resistance [5]. Currently, advanced aircraft engines commonly employ single-crystal superalloy turbine blades with TBC [6,7]. The single-crystal substrate–TBC system employed in turbine blades represents one of the most intricate coating systems in the field of high-temperature materials engineering to date. Currently, the most extensively utilized TBC systems are typically composed of four key components, namely the ceramic topcoat (TC), thermally grown oxide layer (TGO), bond coat (BC), and high-temperature alloy substrate [8,9]. The differences in composition and phase structure between the single-crystal substrate and the coating lead to discontinuities in physical and mechanical properties at the interface. These discontinuities inevitably have a significant impact on the interfacial compatibility between the substrate and the coating. During service, a weak interfacial zone with poor load-bearing capacity is readily formed at the single-crystal substrate-coating interface, often accompanied by the formation of numerous micropores within the bond coat. This markedly reduces the interfacial compatibility of the substrate-coating system, resulting in severe performance degradation, accelerated coating spallation, and even substrate failure. Such issues have become a major concern for the service life of coated single-crystal turbine blades. Therefore, investigating the microstructural evolution and failure behavior of the thermal barrier coating (TBC) system during service, and further elucidating the evolution of the TBC microstructure under high-temperature conditions, is critical for understanding the failure mechanisms of the TBC system.

In recent years, the third-generation nickel-based single-crystal superalloy DD9 has been extensively studied in China [10,11,12]. DD9 is a domestically developed third-generation nickel-based single-crystal superalloy with independent intellectual property rights and low production costs [13]. Compared to the widely used DD6 alloy in the hot-end components of gas turbines in aviation engines [14,15], DD9 offers an improved temperature resistance of approximately 30 °C. The matrix phase of the nickel-based single-crystal superalloy is γ-Ni, which is solid-solution strengthened with elements such as Co, Cr, Mo, W, and Ta, enhancing the alloy’s high-temperature performance and preventing localized hot corrosion [16,17,18,19]. The γ′-Ni_3_Al phase serves as the strengthening phase, with Al being the primary element for the formation of the γ′ phase, contributing to precipitation strengthening. In addition, Al and Cr are also crucial elements for oxidation resistance and corrosion prevention [20,21]. The BC layer is a critical component of the TBC system, as it helps mitigate the thermal mismatch stresses between the TC and the metal substrate, while also protecting the substrate from oxidation [22]. Typically, the elemental composition of the BC layer reflects that of the metal substrate, primarily consisting of the γ phase of Ni and the β phase of Al [23,24,25].

Although the BC layer shares similar elements with DD6 alloy, there are still differences in their elemental compositions. During extended high-temperature operation, the chemical potential gradients drive interdiffusion, leading to microstructural changes and the formation of an interfacial zone [26]. The interfacial zone formed between the bond coat (BC) layer and the substrate includes the interdiffusion zone (IDZ), the secondary reaction zone (SRZ), and the topologically close-packed (TCP) phase. In recent years, the formation mechanisms of the interfacial region and the precipitation behavior of the TCP phase have been extensively studied [27,28]. The IDZ is the forefront of the interdiffusion region in the alloy, and its expansion depends on the direction of element diffusion and the duration of high-temperature exposure [29]. Beneath the IDZ, the SRZ forms, which is a region characterized by substrate recrystallization and the presence of high-density, fine TCP phases [30,31]. Particularly, when large amounts of TCP phases containing elements such as Re, Mo, and W precipitate, the performance of the high-temperature alloy substrate deteriorates rapidly [32]. Studies have shown that the interfacial region has an adverse impact on the service performance of the TBC system [33,34].

There is limited research on the interfacial region based on real turbine blade coating systems from aircraft engines [35,36]. However, due to the high-temperature environment, the formation of the TGO between the BC and TC layers leads to significant changes in the elements of the BC [37,38], which in turn affects the interdiffusion of elements between the BC layer and the metal substrate, altering the formation mechanisms and evolutionary behaviors of the interfacial region. For TBC systems with a thinner BC layer, such as those used in aircraft engines, this effect becomes even more pronounced. Therefore, it is crucial to investigate the formation mechanisms and evolutionary behaviors of the interfacial region in actual service coating systems.

Significant progress has been made in the study of the formation and evolution mechanisms of microstructural features such as the recrystallized layer of the interfacial region, IDZ, and SRZ. Walston et al. [39] were the first to identify the SRZ and pointed out that the SRZ is a key factor in reducing the structural stability of single-crystal superalloys. Suzuki et al. [40] analyzed the formation mechanisms and phase composition of the SRZ. Liang et al. [36] conducted experimental studies on the diffusion behavior in single-crystal superalloy-coating systems, investigating the variation patterns of key elements in the microstructure. They found that oxidation time and temperature significantly influence the microstructural evolution of the interfacial region [30,35]. Due to the unique microstructure of the single-crystal substrate, the SRZ formed exhibits anisotropy [41,42]. Building on this foundation, researchers both domestically and internationally have conducted more in-depth studies on the formation and microstructural evolution of the interfacial region in single-crystal substrate-coating systems, based on different material systems and experimental environments [43,44]. It is evident that current research is limited to experimental studies on the formation and evolution of the microstructure in the interfacial region of single-crystal substrate-coating systems. Theoretical analyses are confined to qualitative explanations of diffusion mechanisms, lacking deeper mechanistic insights.

Currently, the development of nickel-based single-crystal alloys is advancing rapidly abroad, with the temperature tolerance of single-crystal superalloys gradually improving. In contrast, domestic research and development of single-crystal superalloys is relatively recent, and the R&D capabilities are still somewhat limited. In recent years, domestic research and development of nickel-based single-crystal superalloys has made breakthrough progress, with the successful development of third-generation (DD33, DD9) and fourth-generation (DD91, DD15) nickel-based single-crystal superalloys. Among these, the third-generation DD9 alloy exhibits excellent comprehensive performance and has been extensively studied by numerous researchers. Li et al. [13] investigated the mechanical properties of DD9 at different temperatures and found that its yield strength at high temperatures offers a significant advantage. Compared to other single-crystal superalloys, the durability of DD9 is notably superior to that of the second-generation single-crystal superalloy DD6, as well as other foreign second-generation single-crystal superalloys. Shi et al. [45] studied the high-temperature corrosion resistance of DD9 at 900 °C and found that the DD9 single-crystal superalloy exhibits excellent resistance to hot gas corrosion, with its corrosion rate following a parabolic law over time. Zhang et al. [46] investigated the microstructural evolution and failure mechanisms of DD9 under tensile and compressive creep conditions at 1100 °C and 140 MPa. The study found that DD9 exhibited N-type raft formation during tensile creep and P-type raft formation during compressive creep. During tensile creep, the γ′ phase gradually coarsened into a lamellar plate structure, while in compressive creep, the γ′ phase coarsened into a rod-like structure. Furthermore, the raft formation rate was higher in tensile creep than in compressive creep. Although scholars both domestically and internationally have conducted multidimensional research on DD9 nickel-based single-crystal superalloy, there is still a lack of sufficient studies on its performance evolution under high-temperature oxidation environments. Considering its long-term operation under high temperature and pressure, high-temperature oxidation damage cannot be overlooked, as it significantly affects the alloy’s properties. Therefore, a thorough investigation into the high-temperature oxidation behavior of the DD9-TBC system is of great importance for revealing its damage failure mechanisms and establishing a life assessment model.

This study, based on the thermal load data from characteristic regions of turbine blades in actual service, conducts isothermal oxidation experiments on DD9-TBC systems at 1050 °C. The research investigates the damage failure mechanisms of the single-crystal superalloy-coating system and compares the isothermal oxidation behaviors of DD6 and DD9 single-crystal materials. The findings provide valuable insights for understanding the damage evolution and life assessment of coated turbine blades in high-temperature environments.

## 2. Materials and Experiments

### 2.1. Materials Preparation

**Figure 1:**
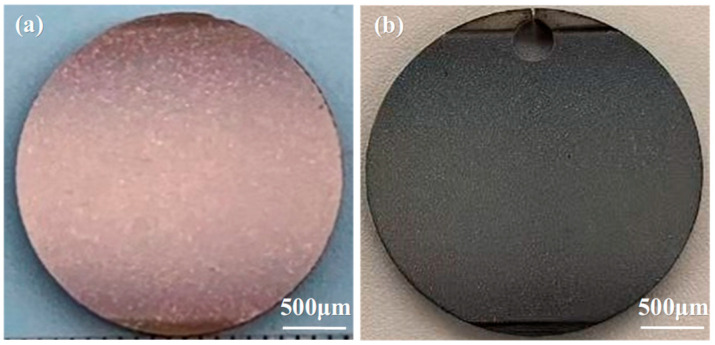
Single crystal wafer specimens with YSZ/NiCoCrAlYHf thermal barrier coatings: (**a**) DD9, and (**b**) DD6.

As shown in Figure 1a, the DD9 single crystal wafer with thermal barrier coating is presented. DD9 is a third-generation single-crystal superalloy, which is prepared by directional solidification technology. Table 1 lists the nominal composition of the material. Prior to the coating preparation, the substrate samples were subjected to surface treatment and activation according to GB 11373-89 [47] “General Principles for Surface Pre-treatment of Thermal Spray Metal Components. A NiCoCrAlYHf coating with a thickness of 20 ± 10 μm, was deposited by arc ion-plating (DH-15, Beiyu Vacuum Technology, Shenyang, China). The arcvoltage was 10~30 V, and arc current was 550~750 A. Following deposition, the coating sample underwent a diffusion process at a temperature of 900 °C for a duration of 2 h (<6 × 10^−3^ Pa) to enhance the compactness and adherence strength [48,49]. The ceramic coating and bond coat materials are YSZ and NiCoCrAlYHf, respectively. The ceramic layer was deposited using an UE204B electron beam physical vapor deposition (EB-PVD) system, with 8 wt.% Y_2_O_3_ partially stabilized ZrO_2_ as the ceramic material. This ceramic layer was applied onto the bond coat, with the YSZ ceramic coating having a thickness of 60 ± 10 μm. For comparative studies, high-temperature oxidation experiments were also conducted on DD6 specimens with thermal barrier coatings under identical conditions. The DD6 single crystal wafer with a thermal barrier coating is shown in Figure 1b.

### 2.2. Oxidation Experiments

The Φ20 × 2 mm circular samples were cut into 4 × 4 mm square specimens using a wire cutting machine (STX-202A, Shenyang, China) and then subjected to polishing. The DD9 single crystal wafer specimens were cleaned using an ultrasonic cleaner and subsequently dried in an oven. The experimental conditions were static atmospheric environment. The single crystal-coating system specimens were placed in a box-type resistance furnace for isothermal oxidation. The furnace was heated to 1050 °C, and the oxidation time was initiated upon reaching this temperature. After the specified experimental time, the specimens were removed, naturally cooled to room temperature, and then subjected to subsequent analysis and characterization.

### 2.3. Materials Characterization

After cutting, the specimens were prepared for scanning electron microscopy (SEM) imaging using a mounting and polishing machine (MoPao2, Laizhou, China). They were ground and polished with sandpapers of 400, 800, 1500, and 3000 grit, followed by polishing with 1, 0.5, and 0.25 μm polishing agents. After polishing, the specimens were cleaned for 10 min using an ultrasonic cleaner. Due to the low conductivity of the ceramic layer, the surface was coated with a gold sputter before observation. The surface and cross-sectional morphology of the as-prepared and isothermally oxidized specimens were observed and characterized using an EVO-10 tungsten filament scanning electron microscope (SEM, ZEISS EVO 10, Oberkochen, Germany). Elemental composition and distribution were analyzed using an energy-dispersive X-ray spectrometer (EDS, ZEISS EVO 10, Germany).

## 3. Oxidation Behavior of the DD9 Superalloy-Coating System

### 3.1. Microstructure of the As-Prepared DD9 Superalloy-Coating System

**Figure 2:**
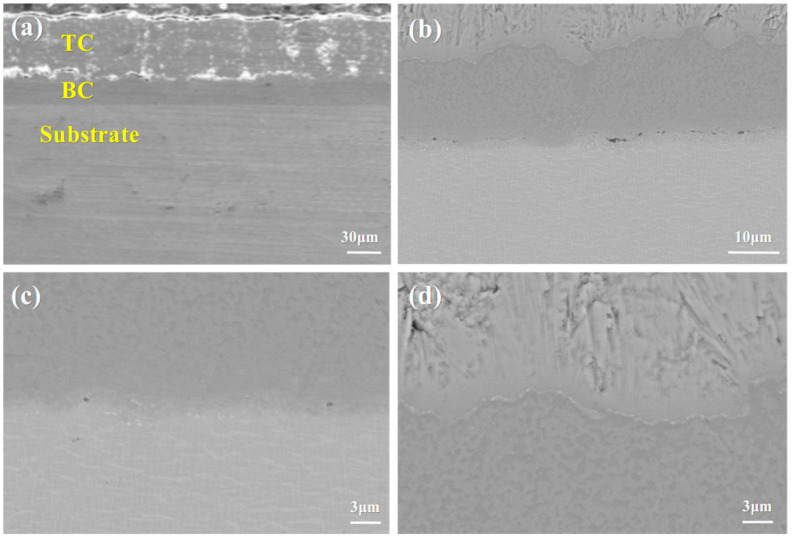
(**a**) Cross-sectional microstructure of the as-prepared DD9 single crystal-coating system, (**b**) local magnified image of the bond coat-substrate interface, (**c**) local magnified image of the BC-Sub interface region, and (**d**) local magnified image of the YSZ-BC interface region.

Figure 2 shows the SEM images of the cross-section of the as-deposited YSZ/NiCoCrAlYHf thermal barrier coating on the circular specimens, captured at different magnifications. Further characterization of the cross-sectional morphology of the as-deposited coating reveals that the thermal barrier coating consists of three layers: the ceramic layer (YSZ), the metallic bond coat (NiCoCrAlYHf), and the metal substrate (DD9). An increase in the roughness of the bond coat enhances the adhesion between the ceramic layer and the metallic bond coat. However, excessive roughness may lead to localized stress concentration, which can accelerate premature failure of the thermal barrier coating. Due to the characteristics of the EB-PVD technique, the ceramic layer of the thermal barrier coating exhibits a columnar structure, with some columnar gaps present. This is consistent with the columnar gaps observed on the surface. A localized magnified observation of the metallic bond coat is shown in Figure 2b. The metallic bond coat is composed of two phases: a light gray phase and a dark gray phase. Both phases are made up of Ni, Co, Cr, and Al elements, with the primary difference being the varying concentration of Al.

**Figure 3:**
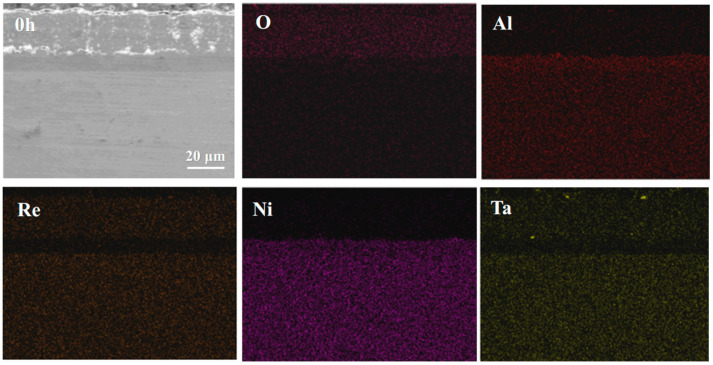
The EDS spectra of the main elements in the as-received specimen.

**Figure 4:**
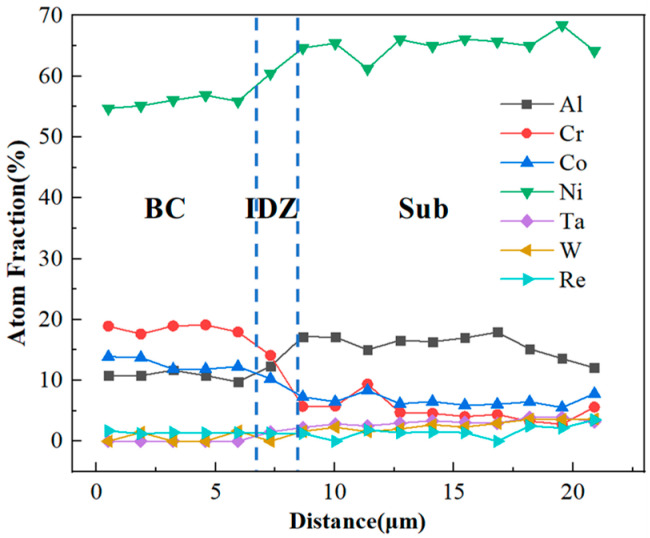
Elemental distribution at the bond coat-substrate interface region.

As shown in Figure 2c, an interdiffusion zone (IDZ) exists at the interface between the NiCoCrAlYHf coating and the DD9 substrate. This is due to the presence of a small amount of oxygen during the heat treatment process. Under low temperature and short duration conditions, a sufficient amount of Al from the coating diffuses primarily to form the IDZ rather than the TGO layer. Figure 2d shows the local magnified image of the YSZ-BC interface region. It can be observed that the YSZ exhibits EB-PVD columnar grain structure, with the columnar gaps oriented perpendicular to the interface, providing a pathway for oxygen diffusion [50,51]. The undulating structure of the BC surface increases the interface contact area, enhancing the coating’s bonding strength through mechanical interlocking. However, this rough interface may lead to localized stress concentration, making it a potential site for the initiation of interface cracking in the later stages of oxidation. Additionally, no obvious initial oxidation layer was observed at the interface, indicating that the YSZ-BC interface of the as-prepared coating is still in a chemically inert state, providing the initial interface conditions for the in situ formation of the TGO during subsequent oxidation. Compared to other substrate elements, the coating contains higher levels of Co and Cr. Figure 3 shows the EDS spectra of the main elements in the as-received specimen. EDS line scan analysis of the cross-section shows that the elemental composition is consistent with that of the target material, as shown in Figure 4. During the heat treatment, the thickness of the original IDZ that has already formed between the coating and the substrate is approximately 2.38 ± 0.37 μm. Figure 2 shows the cross-sectional microstructure of the DD9 superalloy-coating system, clearly presenting the interface morphology and interlayer bonding state of the TC layer, BC layer, and substrate; combined with the elemental distribution results in Figure 4 (the enrichment characteristics of elements such as Zr, Y, Ni, and Al in each layer can be clearly observed), it further verifies that the composition of the coating system is consistent with the design expectations.

### 3.2. Microstructural Evolution of the Single Crystal-Coating System

**Figure 5:**
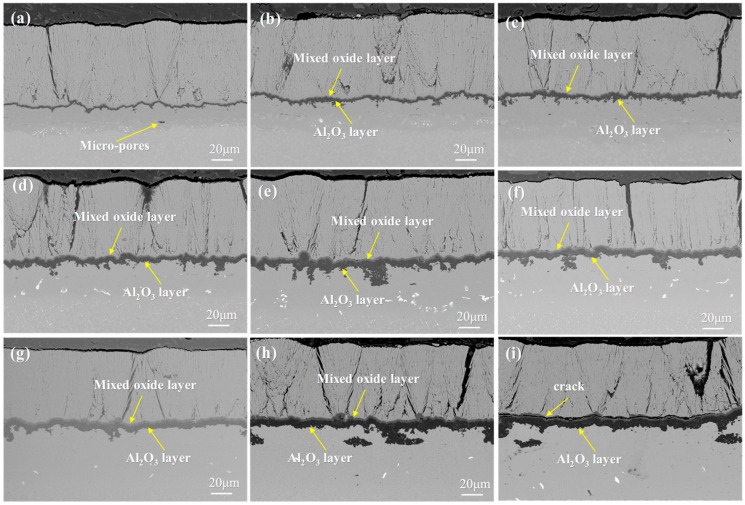
Microstructure of the single crystal DD9-coating system after oxidation at 1050 °C: (**a**) 10 h, (**b**) 50 h, (**c**) 100 h, (**d**) 200 h, (**e**) 300 h, (**f**) 500 h, (**g**) 800 h, (**h**) 1000 h, and (**i**) 1500 h.

Figure 5 shows the microstructural morphology of the single crystal-coating system after oxidation. It is clearly observed that the thermal barrier coating, fabricated by the EB-PVD method, exhibits a typical columnar grain structure. The columnar grains vary in size and grow in a staggered manner, with the upper layers being coarser and more porous, while the lower layers are denser and more continuous. Distinct vertical gaps are present between the columnar grains, which reduce the thermal insulation performance of the coating but improve its strain tolerance.

During the initial stages of oxidation, Al ions within the BC layer diffuse toward the TC layer. Simultaneously, oxygen molecules diffuse inward through the inherent columnar gaps of the TC layer. At the TC/BC interface, these diffusing Al ions react with the inward-diffusing oxygen, ultimately leading to the formation of TGO (as illustrated in Figure 4). As the oxidation reaction proceeds further, the columnar gaps in the TC layer gradually widen, and horizontal cracks start to initiate and propagate within the TC layer. The columnar gaps expand, leading to the formation of vertical cracks. Small pores and interface cracks emerge at the TC/TGO interface. As these interface cracks propagate, they eventually cause the coating to delaminate and spall. The horizontal cracks in the TC layer continue to expand, ultimately leading to the spallation of the TC layer.

**Figure 6:**
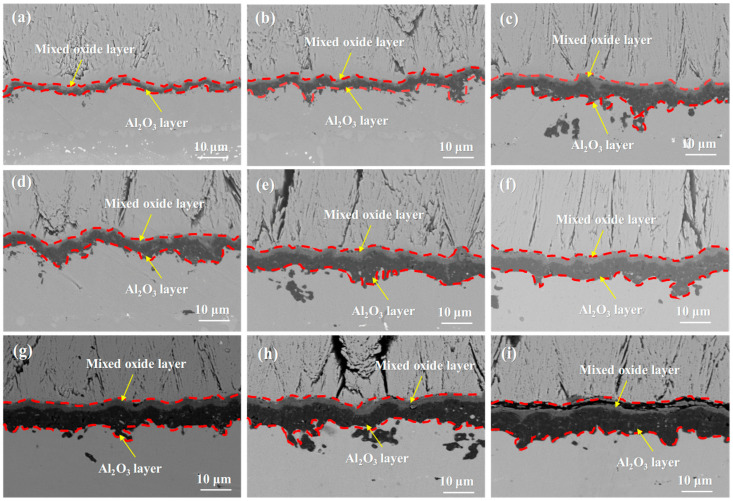
TGO morphology of the single crystal DD9-coating system after oxidation at 1050 °C: (**a**) 10 h, (**b**) 50 h, (**c**) 100 h, (**d**) 200 h, (**e**) 300 h, (**f**) 500 h, (**g**) 800 h, (**h**) 1000 h, and (**i**) 1500 h.

Figure 6 shows the SEM images of the single crystal-coating system after isothermal oxidation at 1050 °C for 10 h, 50 h, 100 h, 200 h, 300 h, 500 h, 800 h, 1000 h, and 1500 h. It can be observed that the thickness of the TGO varies at different locations, and as the oxidation time increases, the fluctuation of the TGO becomes more pronounced. The TGO evolves from a uniform dark gray structure into a bilayer structure, with the upper layer being light gray and the lower layer remaining dark gray. After 100 h of isothermal oxidation, voids appeared at the interface between the ceramic layer and the TGO. After 300 h of oxidation, pores began to form within the TGO. No cracks were observed in the TGO after 500 h. After 800 h, the TGO structure continued to develop, but cracks had still not formed. After 1000 h, cracks started to appear within the TGO. By 1500 h, the cracks within the TGO region had further propagated.

Comparison observations indicate that the TGO layer of DD9 maintains better structural integrity after prolonged oxidation compared to DD6, with a higher density of the Al_2_O_3_ scale. This is attributed to the presence of Re in DD9, which suppresses the migration of oxide grain boundaries. The enrichment of Re reduces the grain growth kinetics of Al_2_O_3_, thereby minimizing the formation of grain boundary porosity. In contrast, the mixed oxide layer in DD6, influenced by the weaker interfacial bonding of Ta, is more prone to microcracking, resulting in slightly poorer integrity of the TGO layer after the same oxidation period.

During the initial stages of oxidation, the columnar gaps provide increased pathways for oxygen infiltration. The Al in the bond coat is exposed to oxygen and rapidly oxidizes, leading to a fast growth of the oxide layer. However, as the oxide layer forms, it impedes further oxygen penetration, resulting in a slowdown of the TGO growth rate [52]. As time progresses, the oxide layer may continue to grow and fracture, exposing the coating to more of the oxidizing environment. This can lead to further oxidation and transformation of the coating material, increasing the generation and accumulation of surface stresses in the coating. The single crystal-coating system can experience stress due to various factors, including stress concentrations during the manufacturing process, temperature fluctuations, physical loading, and changes during the oxidation process. When these stresses exceed the material’s strength limits, cracks may form within the coating. As oxidation time increases, changes in the structure and properties of the coating material may occur, thereby increasing the likelihood of stress buildup within the coating and raising the risk of crack formation [53].

**Figure 7:**
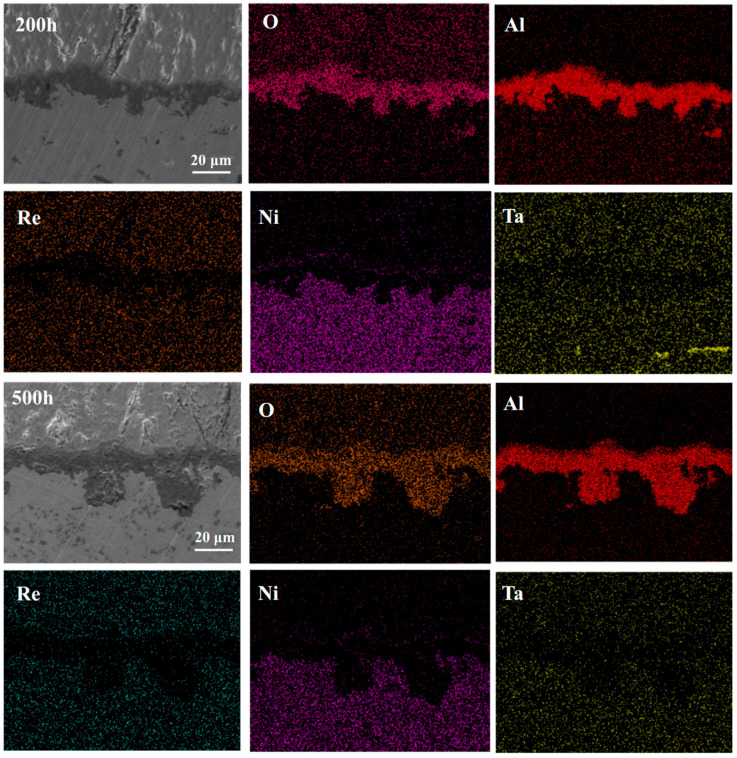
Elemental distribution maps of the TGO after oxidation of the single crystal DD9-coating system at 1050 °C for 200 h and 500 h.

Combining Figure 6 and Figure 7, it can be seen that during the initial stages of oxidation, the primary component of the TGO is Al_2_O_3_ [54,55]. It is generally believed that the formation of Al_2_O_3_ occurs primarily through the inward diffusion of oxygen along the TGO grain boundaries. When the TGO grains are small, Al^3+^ ions may also diffuse outward via lattice diffusion or bulk diffusion, reacting with oxygen ions at the TC/TGO interface to form Al_2_O_3_. After 10 h of oxidation, the TGO exhibits a distinct bilayer structure, with the upper layer containing a significant amount of mixed oxides such as Cr_2_O_3_, NiO, and spinel [56].

The layered structure of the TGO arises from the staged evolution of the oxidation process. In the initial stage, Al in the bond coat selectively oxidizes to form a thermodynamically stable Al_2_O_3_ layer. As oxidation progresses, elements such as Ni and Cr diffuse outward through the oxide grain boundaries, forming a mixed oxide layer above the alumina layer. Increased temperature accelerates the diffusion rate of these elements, leading to the premature appearance of the layered structure. The growth of the TGO follows a typical parabolic law, which is consistent with the oxidation kinetics controlled by ion diffusion. In the initial stages, the rapid consumption of Al promotes the rapid thickening of the oxide layer. However, as the Al content decreases and the oxide layer acts as a diffusion barrier, the growth rate gradually slows down in the later stages.

A comparison reveals that the delamination of the TGO layer in DD6 occurs slightly earlier than in DD9, which is associated with the faster diffusion of Cr in DD6. In DD6, the concentration gradient of Cr between the bond coat and the substrate is greater, facilitating its migration to the surface through TGO grain boundaries to form mixed oxides. In contrast, the presence of Re in DD9 reduces the diffusion coefficient of Ni, thereby delaying the formation of the mixed oxide layer and causing the delamination process to be relatively retarded.

### 3.3. Isothermal Oxidation Kinetic Model of the DD9 Superalloy-Coating System

**Figure 8:**
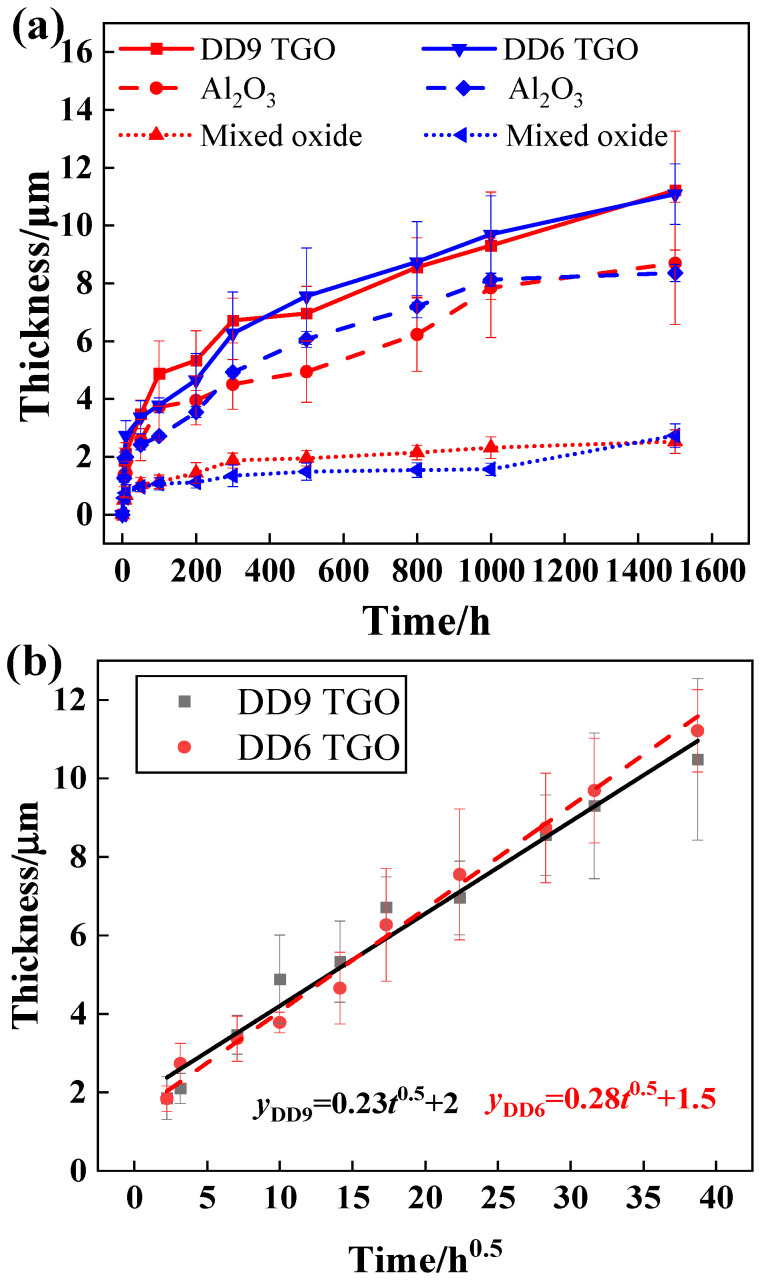
TGO growth kinetics curve of the single crystal-coating system after oxidation at 1050 °C: (**a**) thickening curves for oxidation time, and (**b**) thickening coefficient for oxidation time.

Figure 8 shows the kinetic curves of DD6 and DD9 single crystal substrates with coatings after oxidation at 1050 °C for 1500 h. Within the first 50 h, both coated single crystal substrates exhibit a significant weight gain, after which the weight gain rate slows down considerably. Since the coating elements of both substrates are similar, the TGO thickness generated is also similar. However, DD9 has slightly more elemental differences with the coating, resulting in a lower TGO thickness in the later stages of oxidation compared to DD6. To describe the evolution of the TGO thickness during isothermal oxidation, the TGO images from three different regions were measured using Image J 1.47, and the average TGO thickness was obtained. The TGO thickness was calculated using the following formula:

(1) $$h_{av}=h_{Al}+h_{mix}$$

To clarify the oxidation process, the growth curve of the TGO follows a parabolic fit [57], which can be described by the following equation:

(2) $$h_{av}=k_{p}t^{0.5}+c$$

where $h_{av}$ represents the average TGO thickness from experimental data, $k_{p}$ is the oxidation rate constant, t is the oxidation time, n is the exponent, and *c* is a constant.

By substituting the data into the formula for fitting, the following results were obtained:

(3) $$h_{DD9}=0.23t^{0.5}+2$$

(4) $$h_{DD6}=0.28t^{0.5}+1.5$$

## 4. Microstructure Evolution of the DD9 Superalloy-Coating Interface

Due to the mutual diffusion between the coating and the substrate, under high temperature and stress, it is easy to form an IDZ and even an SRZ. The SRZ below the IDZ consists of a three-phase transformation product, including the γ phase, γ′ phase, and TCP phases. The morphology of the SRZ is closely related to the composition of the high-temperature alloy. Refractory elements such as W, Mo, and Re promote the precipitation of TCP phases within the high-temperature alloy, thereby accelerating the formation of the SRZ. The formation of both the IDZ and SRZ is detrimental to the mechanical properties of the nickel-based single crystal superalloy.

### 4.1. Microstructural Evolution of the IDZ

**Figure 9:**
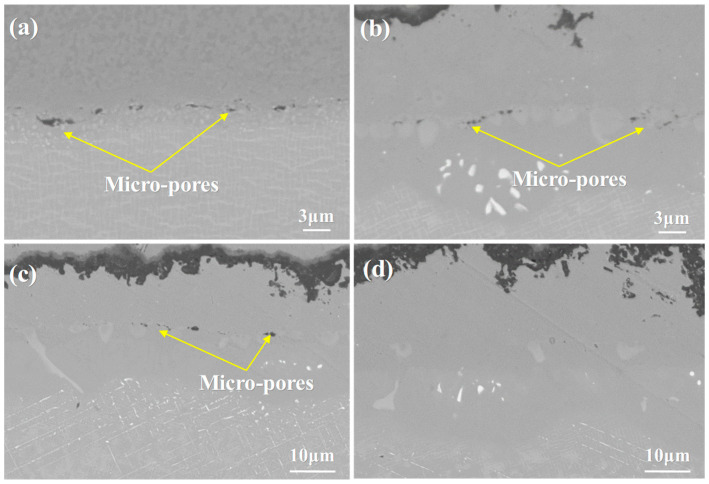
Microstructural images of the porosity at the interface region of the single crystal DD9-coating system after oxidation at 1050 °C: (**a**) Original sample, (**b**) 10 h, (**c**) 50 h, and (**d**) 100 h.

Kirkendall voids refer to small-sized cavities that tend to form in metal materials, particularly during the interdiffusion process between different metal components. Specifically, when two metals with distinct diffusion rates undergo mutual diffusion, a significant number of vacancies are generated in the metal phase with the higher diffusion rate. Over time, these generated vacancies tend to migrate and aggregate at specific sites, eventually resulting in the formation of Kirkendall voids. This phenomenon is named after the Kirkendall effect, which describes the imbalance in diffusion rates between the two metals during the interdiffusion process, leading to the formation of these voids. The formation of Kirkendall voids can lead to a reduction in the strength and corrosion resistance of metal materials. Therefore, it is important to pay attention to avoiding the formation of Kirkendall voids in the design and manufacturing of metal materials. This can be achieved by carefully controlling the diffusion rates of different metals, selecting appropriate alloying elements, and optimizing processing conditions to minimize the occurrence of vacancy defects [58,59,60]. At the same time, the interdiffusion between the coating and substrate can reduce the number of visible porosity defects. When the coating material and the substrate material diffuse into each other, they help fill the tiny voids and bubbles on the coating surface and within the coating. As a result, the density of the coating increases, and the number of defects is reduced. This process can improve the overall integrity and performance of the coating, enhancing its mechanical properties and resistance to environmental degradation. This interdiffusion process leads to a continuous reduction in visible porosity defects, thereby improving the performance and quality of the coating. As shown in Figure 9, a large number of voids appear at the interface of the original sample. With increasing oxidation time, the porosity between the coating and substrate gradually decreases. This phenomenon suggests that interdiffusion enhances the coating-substrate bond and reduces defects, ultimately leading to better coating integrity and durability under high-temperature conditions.

**Figure 10:**
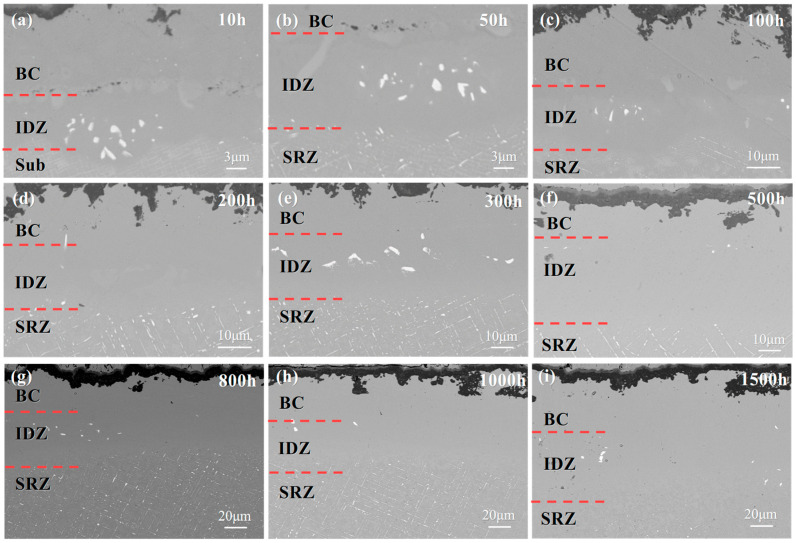
Microstructural evolution of the interface region in the single crystal DD9-coating system after oxidation at 1050 °C: (**a**) 10 h, (**b**) 50 h, (**c**) 100 h, (**d**) 200 h, (**e**) 300 h, (**f**) 500 h, (**g**) 800 h, (**h**) 1000 h, and (**i**) 1500 h.

Figure 10 shows the cross-sectional morphology of the NiCoCrAlYHf coating/DD9 after isothermal oxidation at 1050 °C for nine different durations. As shown in Figure 9a, after 10 h of isothermal oxidation, the β phase in the BC layer is completely diffused and consumed. A light gray phase band appears between the BC layer and the substrate. EDS analysis reveals that this region is rich in Cr [61,62]. Due to the different diffusion fluxes, Kirkendall voids are present in the Cr-rich region. Below the IDZ, a white precipitate phase is observed. As shown in Figure 10b, after 50 h of isothermal oxidation at 1050 °C, an SRZ forms between the IDZ and the single crystal substrate. A fine, granular TCP phase is present between the IDZ and SRZ. The SRZ is primarily composed of needle-like and particulate TCP phases. With increasing isothermal oxidation time, both the IDZ and SRZ thicknesses gradually increase. However, the growth of the IDZ thickness is not significant. After 100 h of isothermal oxidation, the thickness of the IDZ stabilizes, while the SRZ continues to grow noticeably.

**Figure 11:**
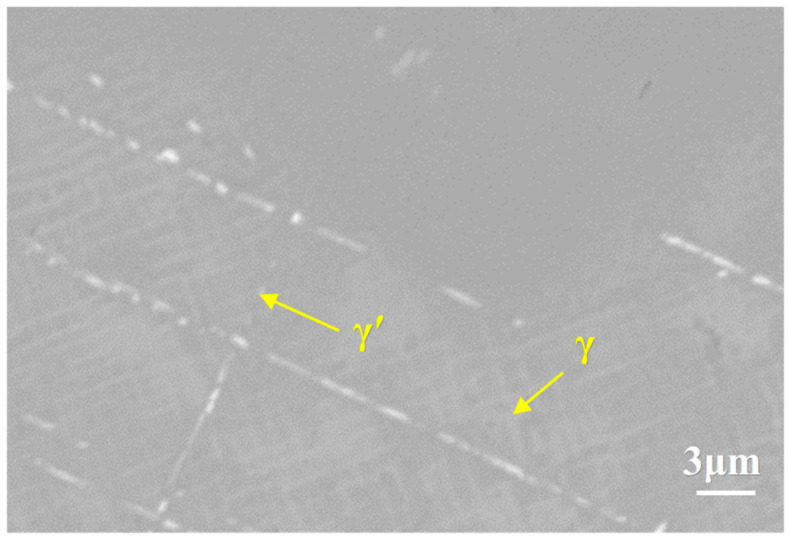
Evolution of the microstructure at the interface of the single-crystal DD9-coating system after oxidation at 1050 °C for 100 h.

As shown in Figure 11, the DD9 single crystal superalloy has a dual-phase structure consisting of γ and γ′ phases. After the NiCoCrAlYHf coating is applied, interdiffusion occurs between the coating and the substrate alloy. This leads to the precipitation of a large number of needle-like TCP phases in the alloy substrate, resulting in a significant reduction in the mechanical properties. The precipitation of needle-like TCP phases is most likely due to the significant differences in Al or Ni content between the NiCoCrAlYHf coating and the substrate alloy. As a result, Al diffuses from the NiCoCrAlYHf coating into the alloy substrate, or Ni diffuses from the alloy into the NiCoCrAlYHf coating. This diffusion process leads to the disruption of the γ phase in the alloy substrate, causing the precipitation of refractory elements and the formation of TCP phases.

The two-phase structure near the TCP phase initially disappears, and the TCP phase precipitates in this region, indicating the presence of a large number of dislocations and vacancies. The precipitation of the TCP phase facilitates element diffusion. Re and other refractory elements continuously accumulate along band-like lines, and the TCP phase precipitates along these band-like regions. The more uniform distribution of Re in the γ′ phase increases the γ/γ′ phase interface area, thereby improving the resistance to dislocation motion at the γ/γ′ interface and enhancing the interface strengthening effect. Re impedes dislocation motion and promotes the precipitation of the TCP phase. The movement of dislocations facilitates the diffusion of Re and the precipitation of the TCP phase. The TCP phase, in turn, accelerates the diffusion of elements and the raft formation of the γ phase.

**Figure 12:**
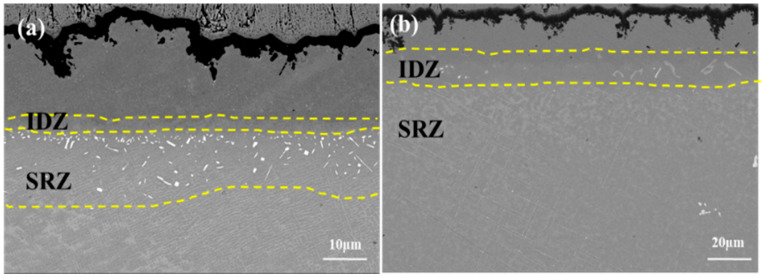
Microstructural morphology of the interface region in the single-crystal DD9-coating system after oxidation at 1050 °C for 100 h: (**a**) DD6, and (**b**) DD9.

A comparison between the DD6 and DD9 substrates reveals that the diffusion behavior in different coating-substrate systems varies, leading to the formation of distinct IDZ and SRZ regions. Additionally, the morphology and quantity of the precipitated TCP phases differ between these systems. As shown in Figure 12a, the interdiffusion and precipitation of the TCP phase in the DD6 substrate results in the formation of needle-like and blocky precipitates. The γ phase around the precipitates undergoes severe rafting, while the SRZ region has not diffused throughout the entire substrate. This leads to a change in the alloy structure, and the precipitation of needle-like TCP phases reduces the mechanical properties of the high-temperature alloy. With the extension of oxidation time, the majority of the TCP phases formed in the substrate do not disappear; instead, they continue to grow further into the alloy matrix. As shown in Figure 12b, the interdiffusion and precipitation of the TCP phase in the DD9 substrate result in the formation of a linear structure, primarily oriented at two distinct angles. The γ phase around the precipitates undergoes severe rafting, and the SRZ region has diffused throughout the entire substrate.

The precipitation of TCP phases follows the electronic vacancy theory. When the element concentration in the interface region reaches a critical value, the increased concentration of electronic vacancies promotes the formation of a TCP structure. These phases tend to exhibit a needle-like morphology, and their elastic modulus significantly mismatches with that of the γ/γ′ dual-phase structure in the substrate. This mismatch leads to the formation of stress concentrations in the interface region. Additionally, the TCP phase, being a hard and brittle phase, acts as a preferential path for crack propagation, reducing the alloy’s fracture toughness and fatigue strength. This phenomenon is directly related to the mechanism where dislocation motion during high-temperature creep deformation is hindered by the presence of TCP phases. The hard and brittle nature of TCP phases disrupts the dislocation movement, making the material more prone to cracking under stress and reducing its overall mechanical performance.

The interdiffusion of elements between the substrate and the bond coat drives the formation and evolution of the interface region. Cr diffuses uphill from the bond coat to the substrate, forming a Cr-enriched IDZ, while Ni diffuses in the reverse direction from the substrate to the bond coat, leading to the decomposition of the γ phase. The rapid expansion of the SRZ is closely related to the precipitation of refractory elements [63]. When the diffusion of Ni weakens the stability of the γ phase, refractory elements such as W and Re precipitate as TCP phases due to the reduced solubility. The presence of specific elements, such as Re, in the DD9 substrate alters the precipitation morphology and distribution of the TCP phase, making it more prone to form a linear structure. In contrast, differences in the elemental composition of different substrate materials, such as DD6, result in significant variations in the morphology of the TCP phase and the growth rate of the interface region.

### 4.2. Elements Interdiffusion Behaviors

**Figure 13:**
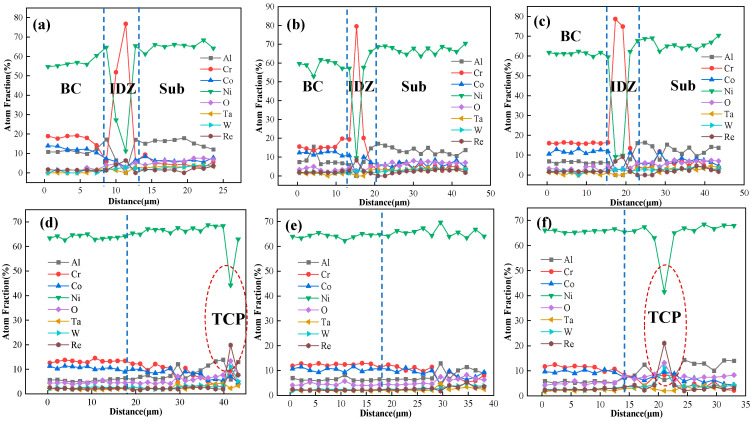
Elemental distribution in the interface region of the single-crystal DD9-coating system after oxidation at 1050 °C: (**a**) 10 h, (**b**) 50 h, (**c**) 100 h, (**d**) 200 h, (**e**) 300 h, and (**f**) 500 h.

The content of Al and Cr in the bond coat is very high to facilitate the rapid formation of a protective Al_2_O_3_ scale on the coating surface. However, when the service temperature reaches 1000 °C or higher, the elemental diffusion between the nickel-based superalloy substrate and the NiCoCrAlYHf coating becomes significant. To more clearly reflect the diffusion behavior of each element during high-temperature service, the EDS point scanning results for the same element at different isothermal oxidation times are compiled. The results are shown in Figure 13. The concentration distribution of each element at different oxidation times shows that diffusion mainly occurs near the interface between the bond coat and the substrate. As the oxidation time increases, the concentration curves gradually become smoother, indicating that the interdiffusion between the coating and the substrate leads to the homogenization of element concentrations.

**Figure 14:**
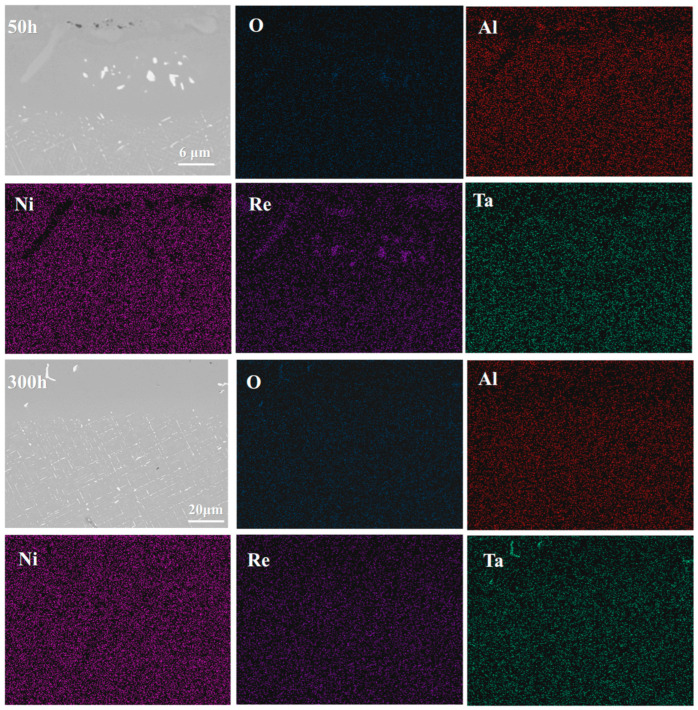
Elemental distribution in the interface region of the single-crystal DD9-coating system after oxidation at 1050 °C for 50 h and 300 h.

In the initial stage of oxidation, Al and Cr elements in the NiCoCrAlYHf bond coat continuously diffuse towards the DD9 substrate, forming an interdiffusion reaction zone. The Cr element accumulates in the interdiffusion zone (IDZ), forming a Cr-enriched layer. This is represented by light gray blocky phases concentrated at the interface region, as shown in Figure 14. However, their concentration decreases as oxidation time increases. With the increase in diffusion time, Al elements continue to form a significant amount of TGO. The Al content in the substrate remains high, and the relatively higher chemical potential leads to the occurrence of reverse diffusion from the substrate towards the coating. Ni, Co, Mo, Ta, W, and Re continuously diffuse towards the NiCoCrAlYHf bond coat. Ni elements progressively diffuse towards the bond coat, forming a secondary reaction zone. In the SRZ, the content of refractory metals (Mo, Ta, W, Re) gradually increases, leading to the continuous precipitation of harmful TCP phases, as shown in Figure 13d,f.

**Figure 15:**
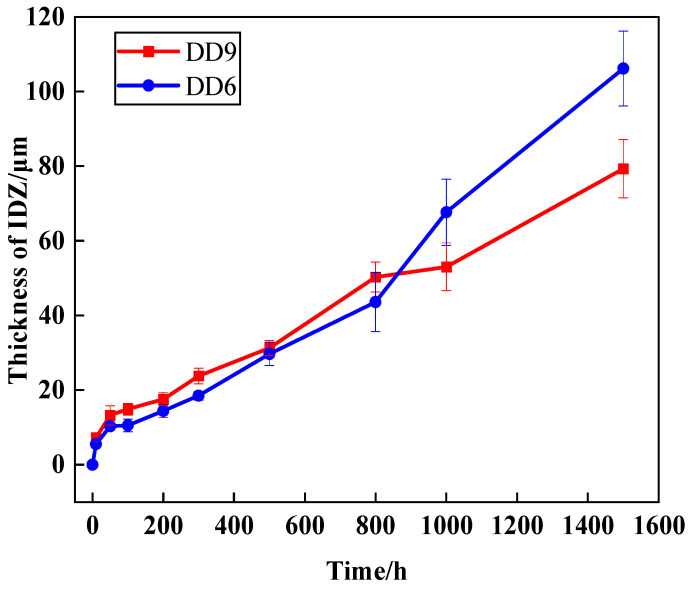
Growth trend of the IDZ formed by interdiffusion in the single-crystal superalloy-coating system after oxidation at 1050 °C for 1500 h, for different substrates.

The IDZ is typically the first region to form after the interdiffusion of Al from the coating and Ni from the substrate. The IDZ forms at the coating/substrate interface and grows into the substrate. The formation of the IDZ can be observed in single-crystal superalloys of various generations with different coatings. Typically, the IDZ may contain phases such as γ′, γ, and β-NiAl. As shown in Figure 15, during the first 800 h, the DD9 substrate exhibits faster diffusion, leading to the quicker formation of the IDZ. However, as the oxidation time increases, the formation of the IDZ in the DD9 substrate gradually becomes slower than that in the DD6 substrate. This may be due to the fact that, in the early stages, the DD6 substrate forms blocky and granular TCP phases tightly adhered to the IDZ/SRZ interface beneath the IDZ. These types of TCP phases hinder elemental diffusion. As oxidation time increases, a large number of blocky and granular TCP phases transform into needle-like TCP phases, which no longer accumulate at the IDZ/SRZ interface, thus eliminating the hindrance to elemental diffusion. On the other hand, in the DD9 substrate, the presence of Re promotes the formation of TCP phases, thereby impeding elemental diffusion.

Additionally, TCP phases precipitate in both substrates, disrupting the γ/γ′ duplex structure and affecting the high-temperature mechanical properties of the single-crystal alloy. This consistent trend provides a common reference for understanding the oxidation behavior of single-crystal superalloy-thermal barrier coating systems. However, the Re element in DD9 delays the growth of the later-stage oxide scale by modulating the morphology of the TCP phases and the diffusion pathways of elements. In contrast, the suppression effect of Ta on TCP phase precipitation in DD6 is relatively weak, making its interfacial zone evolution more sensitive to oxidation time. A comparison between the two alloys indicates that the type and content of refractory elements in the single-crystal substrate are crucial factors in regulating the oxidation stability of the coating system.

The precipitation of TCP phases in the SRZ is related to the electronic structure of alloy elements. When the concentration of elements in the interface region reaches a certain threshold, the increase in electron vacancy concentration promotes the precipitation of TCP phases (such as the μ phase). The precipitation of these phases disrupts the original γ/γ′ duplex structure of the substrate, forming hard and brittle needle-like phases. Due to the significant mismatch in physical properties between the TCP phases and the substrate, stress concentration is introduced at the interface region. This, in turn, reduces the high-temperature strength and fatigue life of the single-crystal alloy, affecting its mechanical performance stability [64].

This study verifies the excellent oxidation resistance stability of DD9 in the medium-to-high temperature region of aero-engines through long-term experiments, analyzes the root causes of high-temperature failure of the system from a microscale perspective, and provides experimental support for formulating the preventive maintenance cycle of turbine blades in engineering practice. Subsequent studies could further focus on the actual service environment of turbine blades, which involves the coupling of “high-temperature oxidation-thermal stress-mechanical load”. Through thermal cycling fatigue experiments combined with multi-physics numerical models, a quantitative correlation between “microstructural evolution-macroscopic failure-life evolution” will be established, which could provide more comprehensive theoretical and technical support for the long-life application of single crystal-TBC systems in high-performance aero-engines.

## 5. Conclusions

In this work, the microstructural evolution and damage failure mechanisms of the DD9 single-crystal-thermal barrier coating system in a 1050 °C high-temperature oxidation environment were investigated. Additionally, the oxidation resistance of DD9 and DD6 alloys was analyzed and compared. The following conclusions can be drawn:

(1) The oxidation mechanisms of DD9 superalloy-coating system and DD6 superalloy-coating system are quite similar. The rapid growth of the TGO leads to the rapid consumption of Al elements in the bond coat, with slower TGO growth in the later stages of oxidation. As oxidation progresses, the TGO develops into two distinct layers: a mixed oxide layer and an Al_2_O_3_ layer. This study provides a theoretical basis for the optimized design of domestic third-generation single-crystal superalloy-thermal barrier coating (TBC) systems.
(2) The substrate has a significant impact on the interdiffusion at the substrate-bond coat interface. As oxidation time increases, both the IDZ and SRZ continue to grow, with the SRZ growing at a faster rate. Line-like TCP phases precipitate within the SRZ, quickly spreading throughout the entire substrate, which can negatively affect the high-temperature mechanical properties of the single-crystal alloy.
(3) In the early stages of oxidation, the growth rate of the TGO and IDZ in DD9 is faster than that in DD6. However, as time progresses, the growth rate of DD6 gradually exceeds that of DD9. This study provides experimental support for the service life evaluation of coated turbine blades.

## Figures and Tables

**Table 1:** Nominal compositions of DD9 and DD6 single crystals and coatings.

	Ni	Co	Cr	Ta	Al	W	Re	Hf	Y	C	Nb	Mo
DD9	Bal	7	3.5	7.5	5.6	6.5	4.5	0.1	0.001	0.008	0.5	2
DD6	Bal	9	4	7	5.7	8	2.2	1	/	/	1	2
BC	Bal	12.5	21	/	10	/	/	0.4	0.3	/	/	/

## Data Availability

The data presented in this study are available on request from the corresponding author due to the relevant data involve the commercial secrets of the research collaborators.

## References

[1] Goucem M. (2024). Influence of the Ambient Temperature on the Efficiency of Gas Turbines. Fluid. Dyn. Mater. Process..

[2] Snyder T., Davis D., McKinney R. (2022). 1 Aero Gas Turbines. Renewable Fuels: Sources, Conversion, and Utilization.

[3] Zhang C., Yang B., Wang Y., Tu G. (2021). Preliminary Research on a High Thrust-to-Weight Ratio of Double-Sided Composite Impeller Microturbine Engine. Int. J. Aerosp. Eng..

[4] Saraçyakupoğlu T. (2024). Energy Savings from New Materials and Processes in Aviation. Sustainable Materials and Manufacturing Techniques in Aviation.

[5] Song J., Qi H., Li S., Shi D., Yang X. (2019). Computational method for the analysis of erosion-induced stress and damage in thermal barrier coatings. Surf. Coat. Technol..

[6] Xia W., Zhao X., Yue L., Zhang Z. (2020). A review of composition evolution in Ni-based single crystal superalloys. J. Mater. Sci. Technol..

[7] Liu Y., Ru Y., Zhang H., Pei Y., Li S., Gong S. (2021). Coating-assisted deterioration mechanism of creep resistance at a nickel-based single-crystal superalloy. Surf. Coat. Technol..

[8] Clarke D.R., Phillpot S.R. (2005). Thermal barrier coating materials. Mater. Today.

[9] Padture N.P., Gell M., Jordan E.H. (2002). Thermal barrier coatings for gas-turbine engine applications. Science.

[10] Shi Z.X., Wang X.G., Liu S.Z., Li J.R. (2016). Rotary bending high cycle fatigue properties of DD9 single crystal superalloy at 800 C. Mater. Mech. Eng..

[11] Wang X., Li J., Yu J., Liu S.Z., Shi Z.X., Yue X.D. (2015). Tensile anisotropy of single crystal superalloy DD9. Acta Metall. Sin..

[12] Yang W.P., Li J.R., Liu S.Z., Shi Z.X., Zhao J.Q., Wang X.G. (2019). Orientation dependence of transverse tensile properties of nickel-based third generation single crystal superalloy DD9 from 760 to 1100 C. Trans. Nonferrous Met. Soc. China.

[13] Li J.R., Liu S.Z., Wang X.G., Shi Z.X., Zhao J.Q. (2016). Development of a Low-Cost Third Generation Single Crystal Superalloy DD9. Superalloys 2016, Proceedings of the 13th Intenational Symposium of Superalloys, Burlington, MA, USA, 11–15 September 2016.

[14] Li J.R., Zhong Z.G., Tang D.Z., Liu S.Z., Wei P., Wu Z.T., Huang D., Han M. (2000). A Low-cost second geneution single crystal superalloy DD6. Superalloys.

[15] Li J.R., Zhao J.Q., Liu S.Z., Han M. (2008). Effects of low angle boundaries on the mechanical properties of single crystal superalloy DD6. Superalloys.

[16] Zhu Z., Basoalto H., Warnken N., Reed R.C. (2012). A model for the creep deformation behaviour of nickel-based single crystal superalloys. Acta Mater..

[17] Li P., Jin X., Zhao J., Lu P., Hu N., Liu D.L., Dong J.M., Fan X.L. (2022). Oxidation behaviors and compressive strength evolution of DD6 Ni-based single-crystal superalloy at 1100 °C. Corros. Sci..

[18] Sumoyama D., Thosin K.Z., Nishimoto T., Yoshioka T., Izumi T., Hayashi S., Narita T. (2007). Formation of a Rhenium-base Diffusion-barrier-coating System on Ni-base Single Crystal Superalloy and its Stability at 1423 K. Oxid. Met..

[19] Suzuki A., Kawagishi K., Yokokawa T., Harada H. (2012). Effect of Cr on microstructural evolution of aluminized fourth generation Ni-base single crystal superalloys. Surf. Coat. Technol..

[20] Zhou L., Mehta A., Cho K., Sohn Y. (2017). Composition-dependent interdiffusion coefficient, reduced elastic modulus and hardness in γ-, γ′- and β-phases in the Ni-Al system. J. Alloy. Compd..

[21] Guo H., Gong S., Khor K.A., Xu H. (2003). Effect of thermal exposure on the microstructure and properties of EB-PVD gradient thermal barrier coatings. Surf. Coat. Technol..

[22] Xie S., Lin S., Shi Q., Wang W., Song C., Xu W., Dai M. (2021). A study on the mechanical and thermal shock properties of MCrAlY coating prepared by arc ion plating. Surf. Coat. Technol..

[23] Kopec M., Kukla D., Yuan X., Rejmer W., Kowalewski Z.L., Senderowski C. (2021). Aluminide thermal barrier coating for high temperature performance of MAR 247 nickel based superalloy. Coatings.

[24] Yang H.Z., Zou J.P., Shi Q., Lin S.S., Dai M.J., Wang D. (2020). Recent advances for interface diffusion behavior in MCrAlY coatings at elevated temperature oxidation. Rare Met. Mat. Eng..

[25] Texier D., Monceau D., Hervier Z., Andrieu E. (2016). Effect of interdiffusion on mechanical and thermal expansion properties at high temperature of a MCrAlY coated Ni-based superalloy. Surf. Coat. Technol..

[26] Sun X., Zhang P., Moverare J., Li H.X., Cui L.Q., Peng R.L. (2023). Impeding the γ′ depletion during the interdiffusion between bond coatings and superalloys via introduction of tantalum in bond coatings. Mater. Des..

[27] Tan X.P., Hong H.U., Choi B.G., Kim I.S., Jo C.Y., Jin T., Hu Z.Q. (2013). Characterization of topologically close-packed phases in secondary reaction zone in a coated CMSX-4 single crystal Ni-based superalloy. J. Mater. Sci..

[28] Bai Z., Li D., Peng H., Wang J., Guo H., Gong S. (2012). Suppressing the formation of SRZ in a Ni-based single crystal superalloy by RuNiAl diffusion barrier. Prog. Nat. Sci..

[29] Yin B., Ni J., Deng P., Li Q., Mao J., Zhang L., Deng C. (2022). Microstructure evolution and interdiffusion of PtAl coated a third generation single crystal superalloy during thermal exposure. Vacuum.

[30] Yang L., Chen M., Wang J., Qiao Y.X., Guo P.Y., Zhu S.L., Wang F.H. (2020). Microstructure and composition evolution of a single-crystal superalloy caused by elements interdiffusion with an overlay NiCrAlY coating on oxidation. J. Mater. Sci. Technol..

[31] Bai B., Guo H., Peng H., Peng L.Q., Gong S.K. (2011). Cyclic oxidation and interdiffusion behavior of a NiAlDy/RuNiAl coating on a Ni-based single crystal superalloy. Corros. Sci..

[32] Zhong J., Liu J., Zhou X., Li S., Yu M., Xu Z. (2016). Thermal cyclic oxidation and interdiffusion of NiCoCrAlYHf coating on a Ni-based single crystal superalloy. J. Alloy. Compd..

[33] Geng L., Zhao W., Ru Y., Pei Y., Li S., Gong S. (2023). Tailoring coating composition for the associated microstructural stability of a single-crystal superalloy: An experimental and simulation study. Corros. Sci..

[34] Zhan X., Wang D., Ge Z., Zhang Y., Dong J., Lou L., Zhang J. (2022). Microstructural evolution of NiCoCrAlY coated directionally solidified superalloy. Surf. Coat. Technol..

[35] Shi L., Xin L., Wang X., Wang X., Wei H., Zhu S., Wang F. (2015). Influences of MCrAlY coatings on oxidation resistance of single crystal superalloy DD98M and their inter-diffusion behaviors. J. Alloy. Compd..

[36] Liang T., Guo H., Peng H., Gong S. (2011). Precipitation phases in the nickel-based superalloy DZ 125 with YSZ/CoCrAlY thermal barrier coating. J. Alloy. Compd..

[37] Vorkötter C., Mack D.E., Guillon O., Vaßen R. (2019). Superior cyclic life of thermal barrier coatings with advanced bond coats on single-crystal superalloys. Surf. Coat. Technol..

[38] Naumenko D., Shemet V., Singheiser L., Quadakkers W.J. (2009). Failure mechanisms of thermal barrier coatings on MCrAlY-type bondcoats associated with the formation of the thermally grown oxide. J. Mater. Sci..

[39] Walston W.S., Ross E.W., Pollock T.M., O’Hara K.S., Murphy W.H. (1995). Nickel-Base Superalloy and Article with High Temperature Strength and Improved Stability. U.S. Patent.

[40] Suzuki A.S., Rae C.M., Hobbs R., Murakami H. (2011). Secondary reaction zone formations in Pt-Aluminised fourth generation Ni-base single crystal superalloys. Adv. Mater. Res..

[41] Wu J., Jiang X., Song P., Wang Y., Dong J.S., Lou L.H. (2020). Anisotropy of interface characteristics between NiCoCrAlY coating and a hot corrosion resistant Ni-Based single crystal superalloy during thermal exposure at different temperatures. Appl. Surf. Sci..

[42] Yin B., Xie G., Lou L., Zhang J. (2020). Effect of Ta on microstructural evolution of NiCrAlYSi coated Ni-base single crystal superalloys. J. Alloys Compd..

[43] Wang R., Gong X., Peng H., Ma Y., Guo H. (2015). Interdiffusion behavior between NiAlHf coating and Ni-based single crystal superalloy with different crystal orientations. Appl. Surf. Sci..

[44] Guo C., Zhou F., Chen M., Wang J., Zhu S., Wang F. (2021). An in-situ formed ceramic/alloy/ceramic sandwich barrier to resist elements interdiffusion between NiCrAlY coating and a Ni-based superalloy. J. Mater. Sci. Technol..

[45] Shi Z.X., Liu S.Z., Wang X.G., Li J.R. (2016). Hot-gas corrosion resistance of DD9 single crystal superalloy. Materials Science Forum.

[46] Zhang Z., Wen Z., Yue Z. (2021). Effects of tensile/compressive creeps on microstructure evolution of nickel-based single crystal superalloys. J. Alloy. Compd..

[47] (1989). The General Principle of Surface Preparation of Metallic Substrate for Thermal Spraying.

[48] Liu D., Mu R., He L., Li S., Yang W. (2023). Failure behaviour of EB-PVD YSZ thermal barrier coatings under simulated aero-engine operating conditions. Surf. Coat. Technol..

[49] Li P., Fan X., Lu P., Wang H., Jin X.C. (2023). Effect of pre-treatment temperatures on the oxidation behaviors and surface roughening mechanisms of NiCoCrAlYHf coating. Corros. Sci..

[50] Xie S., Dai M., Lin S., Shi Q., Song C., Hou H., Qiu W., Wang Y. (2019). Effect of bias voltage on the oxidation resistance of NiCoCrAlYTa coatings prepared by arc ion plating. Corros. Sci..

[51] Wang J., Xiong K., Jin X., Li P., Li Z., Li J., Hou C., Fan X. (2024). Comparative study of temperature-dependent oxidation and interdiffusion behavior on NiCoCrAlYHf-coated nickel-based single-crystal superalloys. J. Mater. Sci..

[52] Wang L., Li Z., Ding K., Deng C., Zhang S.Y., Zheng R.G., Yang L.W., Lin X.P. (2022). Effects of TGO growth on the stress distribution and evolution of three-dimensional cylindrical thermal barrier coatings based on finite element simulations. Ceram. Int..

[53] Xu Q.L., Liu K.C., Wang K.Y., Lou L.Y., Zhang Y., Li C.J., Li C.X. (2021). TGO and Al diffusion behavior of CuAlxNiCrFe high-entropy alloys fabricated by high-speed laser cladding for TBC bond coats. Corros. Sci..

[54] Heuer A.H., Hovis D.B., Smialek J.L., Gleeson B. (2011). Alumina scale formation: A new perspective. J. Am. Ceram. Soc..

[55] Heuer A., Nakagawa T., Azar M., Hovis D., Smialek J., Gleeson B., Hine N., Guhl H., Lee H.-S., Tangney P. (2013). On the growth of Al_2_O_3_ scales. Acta Mater..

[56] Zhou J., Gao W., Liu L., Yi T., Jiang W.X., Wang J., Zhang Y.F., Zhang Z. (2023). In-situ SEM study on fatigue crack behavior of a nickel-based single crystal alloy at 950 °C and 1050 °C. Mater. Charact..

[57] Pei H., Wang J., Li Z., Yao X., Wen Z., Yue Z. (2021). Oxidation behavior of recast layer of air-film hole machined by EDM technology of Ni-based single crystal blade and its effect on creep strength. Surf. Coat. Technol..

[58] Gao F., Nishikawa H., Takemoto T. (2008). Additive effect of kirkendall void formation in Sn-3.5Ag solder joints on common substrates. J. Electron. Mater..

[59] Cho M.G., Kang S.K., Shih D.Y., Lee H.M. (2007). Effects of minor additions of Zn on interfacial reactions of Sn-Ag-Cu and Sn-Cu solders with various Cu substrates during thermal aging. J. Electron. Mater..

[60] Tsai J.Y., Hu Y.C., Tsai C.M., Kao C.R. (2003). A study on the reaction between Cu and Sn3.5Ag solder doped with small amounts of Ni. J. Electron. Mater..

[61] Liang T., Guo H., Peng H., Gong S. (2011). Microstructural evolution of CoCrAlY bond coat on Ni-based superalloy DZ 125 at 1050 °C. Surf. Coat. Technol..

[62] Tian S., Zhang Y., He A., Liu J.H., Zeng S.W., Jiang H.T. (2022). Interdiffusion mechanism at the interface between TiAl alloy and NiCoCrAlY bond coating. Surf. Coat. Technol..

[63] Sun J., Liu J., Chen C., Li J., Wang X., Sun X. (2022). Effect of Ru on γ/γ′ microstructural evolutions and precipitation of TCP phases during thermal exposure at 1100 °C in a single crystal superalloy. Mater. Charact..

[64] Jin H., Zhang J., Zhang Y., Zhang W.Y., Ma S.Y., Mao S.C., Du Y.Q., Wang Z.H., Qin J.Y., Wang Q. (2022). Effects of the orientation relationships between TCP phases and matrix on the morphologies of TCP phases in Ni-based single crystal superalloys. Mater. Charact..

